# Human T-cell leukemia virus type 2 Tax protein induces interleukin 2-independent growth in a T-cell line

**DOI:** 10.1186/1742-4690-3-88

**Published:** 2006-12-02

**Authors:** Rie Kondo, Masaya Higuchi, Masahiko Takahashi, Masayasu Oie, Yuetsu Tanaka, Fumitake Gejyo, Masahiro Fujii

**Affiliations:** 1 Division of Virology, Niigata University Graduate School of Medical and Dental Sciences, 1-757 Asahimachi-Dori, Niigata 951-8510, Japan; 2 Division of Clinical Nephrology and Rheumatology, Niigata University Graduate School of Medical and Dental Sciences, 1-757 Asahimachi-Dori, Niigata 951-8510, Japan; 3 Department of Immunology, Graduate School and Faculty of Medicine, University of the Ryukyus, Uehara 207, Nishihara-cho, Nakagami-gun, Okinawa 903-0215, Japan

## Abstract

**Background:**

While human T-cell leukemia virus type 1 (HTLV-1) is a causative agent of adult T-cell leukemia, HTLV type 2 (HTLV-2) is not associated with this malignancy. Accumulating evidence suggests that Tax, a transforming protein of HTLV-1 or HTLV-2, plays a crucial role in the distinctive pathogenesis of these two infections. We herein examined whether Tax2 by itself has a growth promoting activity in a mouse T-cell line CTLL-2, and compared the activity with that of Tax1.

**Results:**

We found that Tax2 converts the cell growth of CTLL-2 from an interleukin(IL)-2-dependent growth into an independent one. Cyclosporine A, an inhibitor of transcription factor NFAT, inhibited the growth of two out of four Tax2-transformed CTLL-2 cells, but it had little effect on two Tax1-transformed cells. While the HTLV-2-transformed human T-cell lines produce a significant amount of IL-2, Tax2-transformed CTLL-2 cells only produced a minimal amount of IL-2. These results thus suggest that NFAT-inducible gene(s) other than IL-2 play a role in the cell growth of Tax2-transformed CTLL-2 cells.

**Conclusion:**

These results show that HTLV-2 Tax2 by itself has a growth promoting activity toward a T-cell line CTLL-2, and the CTLL-2 assay used in this study may therefore be a useful tool for comparing the activity of Tax2 with that of Tax1 in T-cells, thereby elucidating the mechanism of HTLV-1 specific leukemogenesis.

## Findings

Human T-cell leukemia virus type 1 (HTLV-1) and HTLV type 2 (HTLV-2) are a family of retroviruses, which share around a 70% nucleotide identity and similar biological properties [1-6]. For instance, both HTLV-1 and HTLV-2 can efficiently transform primary human T-cells *in vitro *and establish a life-long persistent infection in humans [7-9]. The clinical outcomes of these two infections are, however, significantly distinctive. While HTLV-1 is etiologically associated with adult T-cell leukemia (ATL), HTLV-2 is associated with only a few cases of variant hairy cell leukemia [5,10-12].

HTLV-1 and HTLV-2 encode a transforming protein Tax1 and Tax2, respectively, which are essential for the transformation of primary human T-cells *in vitro *[13-16]. Accumulating evidence suggests that Tax1 is a factor responsible for the high-oncogenic activity of HTLV-1 relative to HTLV-2 [4,5]. Tax1 and Tax2 have more than 75 % amino acid identities, and they also exhibit strikingly similar functions in infected cells [17,18]. For instance, Tax1 and Tax2 induce the expression of a number of cellular genes through several transcription factor binding sites, such as NF-κB, CREB/ATF, SRF, and AP-1 [4,19-25]. These Tax-inducible cellular genes play a critical role in the persistent infection in host T-cells, including the transformation of human T-cells [24,25], but they alone can not explain the pathogenic differences between HTLV-1 and HTLV-2, since the potencies of these functions are equivalent. On the other hand, recent results identified several differences between Tax1 and Tax2, which are likely to be factors that are responsible for the pathogenic difference of two infections [4,5,26-35]. Therefore, a comparative analysis of Tax1 and Tax2 is a promising approach to identify a key process responsible for HTLV-1 specific leukemogenesis.

**Figure 1:**
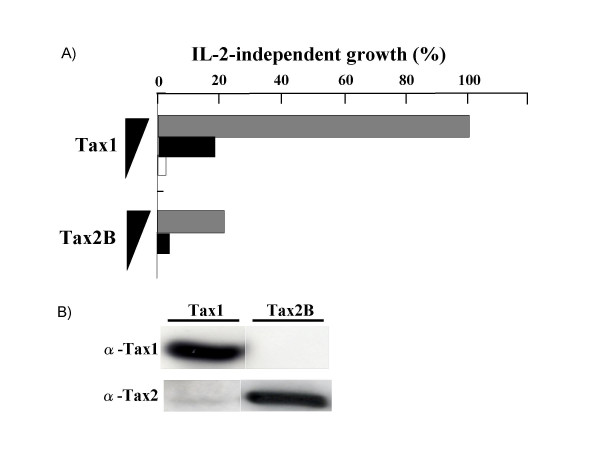
**Tax2 induces the IL-2-independent growth of CTLL-2 cells**. (A) *tax1 *and *tax2B *cDNAs were cloned into the lentivirus vector CSIIEF-RfA which has an elongation factor gene promoter for protein expression in mammalian cells. Lentiviruses encoding Tax1 and Tax2B were produced by the three plasmid cotransfection method in 293T cells derived from an embryo kidney. These lentiviruses were transduced to CTLL-2 cells (4 × 10^5^) in a final volume of 2.0 ml RPMI1640 containing 10% fetal bovine serum (RPMI/10%FBS), 8 μg/ml polybrene (Sigma) and 1 nM recombinant human IL-2 (Takeda). At 48 hours after infection, the infected cells were washed twice with phosphate-buffered saline (PBS), and the serially diluted cells (330/well, 1000/well, 10000/well) were cultured in 96 well plate containing RPMI/10%FBS without IL-2. Four weeks later, the number of wells containing outgrowing cells was counted by light microscopy. IL-2-independent growth (%) was calculated as a ratio of the number of positive wells out of 96 wells. (B) Tax2 proteins in transiently lentivirus-infected CTLL-2 cells were undetectable (data not shown). Therefore, a human T-cell line Jurkat was infected with the lentiviruses encoding Tax1 or Tax2, and 48 hr after the infection, the amount of Tax proteins in Jurkat was measured by a Western blotting analysis. The Western blotting assay was carried out as previously described [37]. The antibodies used were anti-Tax1 monoclonal antibody (Taxy7) [38] and anti-Tax2B polyclonal antiserum, kindly provided by Dr. W.W. Hall (University College Dublin) [39],.

**Figure 2:**
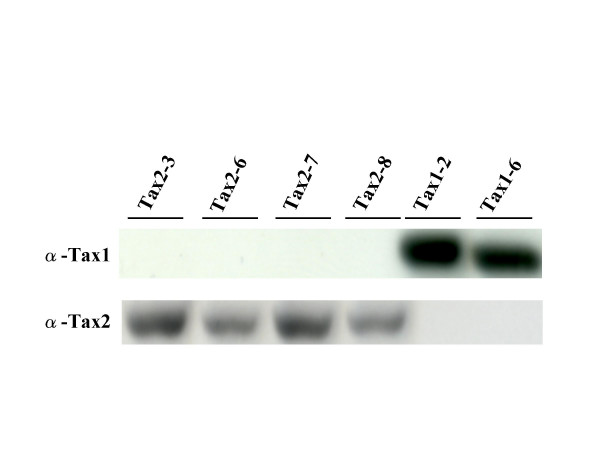
**Expression of Tax protein in transformed cells**. Cell lysates were prepared from the indicated cells, and the amounts of Tax proteins were measured by a Western blotting analysis with either anti-Tax1 or anti-Tax2 antibodies as described in Figure 1 [37].

We previously showed that Tax1 transforms a mouse T-cell line CTLL-2 from an interleukin(IL)-2-dependent growth to an IL-2-independent one, whereas Tax2 can not do so [32,36]. We herein reexamined the transforming activity of Tax2 in CTLL-2 using a lentivirus vector for the transduction of the *tax *gene which is much more efficient than the electroporation method used in a previous experiment. Lentiviruses encoding Tax1 or Tax2 were produced in 293T cells, and these viruses were then infected to CTLL-2 cells in a medium containing IL-2. At 48 hours after infection, the infected cells were cultured without IL-2 in a 96 well plate. Four weeks later, the number of wells containing outgrowing cells was counted by light microscopy. Unlike the previous study, Tax2 transduced with a lentivirus induced the IL-2-independent growth of CTLL-2 cells (Figure 1). A Western blotting analysis using Tax1 and Tax2 antibodies showed that all four Tax2-transformed cell lines expressed Tax2 protein but not Tax1 (Figure 2), thus confirming that the tax2-virus induced the transformation. Like Tax1, these Tax2-transformed CTLL-2 cells continuously grow in the absence of IL-2 for at least three months (data not shown). These results showed that Tax2 therefore induced the IL-2-independent growth of CTLL-2 cells.

**Figure 3:**
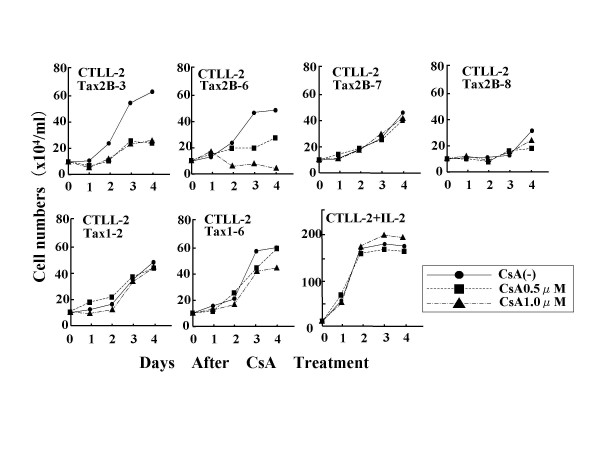
**Growth inhibition of Tax2-transformed cells by cyclosporine A**. IL-2-independent CTLL-2 cells stably expressing Tax1 or Tax2B were seeded at 2 × 10^5 ^cells/well on a 48-well plate and cultured in the presence of either 0.5 μM or 1.0 μM of cyclosporine (Sigma). After culturing for the indicated days, viable cell numbers were counted by a trypan blue dye exclusion method using light microscopy.

**Figure 4:**
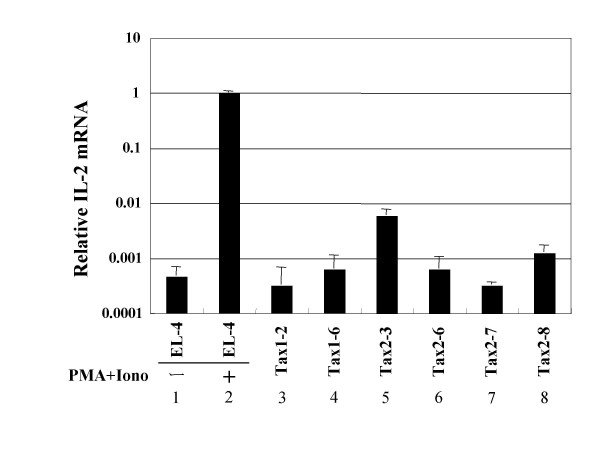
**Minimum expression of IL-2 mRNA in Tax2-transformed cells.** Total RNA was isolated from indicated Tax-transformed CTLL-2 cells (lanes 3–8), or EL-4 T-cell line treated with (lane 2) or without (lane 1) 20 μg/ml phorbol myristate acetate and 1 μM ionomycin for 5 hours using RNAiso reagent, according to the manufacturer's instructions (Takara, Kyoto, Japan), and then total RNA (500 ng) was reverse transcribed using ExScript RT reagent kit (Takara). To quantify the amount of IL-2 RNA, a real-time polymerase chain reaction (PCR), based on SYBR green fluorescence, was performed using SYBR Premix Ex Taq polymerase and Takara real-time Thermal Cycler Dice (Takara). The following primers were used to specifically amplify respective genes: mouse IL-2 gene, 5'-GGAGCAGCTGTTGATGGACCTAC-3' and 5'-AATCCAGAACATGCCGCAGAG-3', mouse glyceraldehyde-3-phosphate dehydrogenase gene used as a control, 5'-TGTGTCCGTCGTGGATCTGA-3' and 5'-TTGCTGTTGAAGTCGCAGGAG-3'.

We previously showed that Tax2 activates IL-2 transcription through the transcription factor NFAT, and such produced IL-2 in some HTLV-2-transformed T-cell lines stimulates their cell growth [37]. To examine whether NFAT plays a role in the Tax2-mediated IL-2-independent growth of CTLL-2, we cultured Tax2-transformed cells with cyclosporine A (CsA), a specific inhibitor of NFAT pathway (Figure 3). Two out of four Tax2-transformed cells showed reduced cell growth in the presence of CsA, while the other two showed little response to CsA treatment. On the other hand, parental CTLL-2 and two Tax1-transformed cells did not show CsA-mediated growth inhibition. These results show that the activation of NFAT by Tax2 stimulates the cell growth of some Tax2-transformed cells, but not Tax1-transformed ones. A real-time polymerase chain reaction with IL-2-specific primers showed that Tax2-transformed CTLL-2 cells minimally expressed IL-2 mRNA, whereas EL-4 T-cell line treated with phorbol myristate acetate and ionomycin produced a significant amount of IL-2 mRNA (Figure 4). We consistently detected IL-2 protein in the culture supernatant of the EL-4 cells treated with the same mitogens, but not those of the Tax2-transformed cell lines (data not shown). These results suggest that NFAT-inducible gene(s) other than IL-2 are thus involved in the Tax2-mediated growth promotion of CTLL-2 cells.

Tax2 has been shown to be essential for HTLV-2-mediated transformation of human T-cells [15]. It, however, remains to be elucidated whether Tax2 by itself has a growth promoting activity toward T-cells like Tax1 [36]. We herein showed that Tax2 can reproducibly convert a mouse T-cell line from an IL-2-dependent growth into an independent one. These results demonstrate that Tax2 by itself without any other viral proteins has a growth promoting activity in T-cells, thus suggesting that this growth promoting activity of Tax2 contributes to HTLV-2-mediated T-cell transformation. Since at least two functions, apoptosis inhibition and cell cycle promotion are both required for CTLL-2 to grow in the absence of IL-2, Tax2 can therefore replace these two functions in CTLL-2.

CsA inhibited the growth of two out of four Tax2-transformed CTLL-2 cells (Figure 3), indicating that NFAT-inducible genes are involved in IL-2-independent growth of these Tax2-transformed cells. These results are consistent with the previous results that CsA inhibited cell growth of some but not all HTLV-2-transformed human T-cell lines [37]. There are at least two explanations for the distinct responses of the Tax2-transformed cells to CsA. Tax2 may have two distinctive activities to induce IL-2-independent growth of CTLL-2 cells. Alternatively, some parental CTLL-2 cells may have genetic or epigenetic change(s) conferring resistance to CsA in Tax2-transformed CTLL-2 cells. In contrast to Tax2, the cell growth of Tax1-transformed cells was little affected by CsA. This finding is also consistent with the result that Tax1 minimally activates NFAT, and thus CsA can not inhibit the cell growth of any HTLV-1-transformed T-cell lines [37].

Unlike the HTLV-2-transformed human T-cell lines sensitive to CsA-mediated growth inhibition, Tax2-transformed CsA-sensitive cells expressed a small amount of IL-2 mRNA (Figure 4). Since there are several NFAT inducible cytokines which promote T-cell growth, such as IL-4 and IL-21, these results indicated that the NFAT-inducible gene(s) other than IL-2 positively regulate the cell growth of the Tax2-transformed cells, thus suggesting that HTLV-2-transformed human T-cells may also utilize multiple NFAT-inducible T-cell growth promoting factors for their growth.

Accumulating evidence suggests that Tax plays a crucial role in the distinctive pathogenesis between HTLV-1 and HTLV-2 [4,5,26,28,29,32,34]. Therefore, further comparative studies of the Tax1 and Tax2 functions in T-cells are expected to advance our understanding of HTLV-1 leukemogenesis. The CTLL-2 assay used in this study is therefore considered to be a useful tool for examining the functions of Tax2 and Tax1 in T-cells, thereby elucidating the mechanism of HTLV-1 specific leukemogenesis.

## Competing interests

The author(s) declare that they have no competing interests.

## Authors' contributions

RK, MH, MT, LX, and YT carried out the establishing the cell lines and the functional analysis of the cell lines. MO, FG, and MF participated in the experimental design, data interpretation, and the writing of the manuscript.
